# Posaconazole-Vincristine Coadministration Triggers Seizure in a Young Female Adult: A Case Report

**DOI:** 10.1155/2012/343742

**Published:** 2012-03-14

**Authors:** Dalia A. Hamdy, Hager El-Geed, Samah El-Salem, Manal Zaidan

**Affiliations:** ^1^College of Pharmacy, Qatar University, P.O. Box 2713, Doha, Qatar; ^2^Al-Amal Hospital, Hamad Medical Corporation, Doha, Qatar

## Abstract

Coadministration of azoles and vincristine has been shown to increase vincristine neurotoxic effects due to the inhibition of cytochrome P450 (CYP) isoform 3A4, for which vincristine is a substrate. Despite the absence of any casual relationship between seizure and coadministration of azoles, few case reports of vincristine-induced seizure have been documented after coadministration of fluconazole or posaconazole in children. In this paper we are reporting the first young female adult who experienced generalized seizure after coadministration of posaconazole and vincristine. The 19-year-old female was diagnosed with acute lymphoblastic leukemia. She started induction phase of Berlin Frankfurt Muenster protocol along with posaconazole 200 mg three times daily as prophylactic antifungal therapy. Five days after the third vincristine dose, she developed generalized seizure accompanied by high blood pressure and SIADH. Her neurological exam/CT scan did not show any abnormality. In conclusion, this study reports a novel finding in the sense that all previous case reports pertaining to posaconazole-vincristine-induced seizure in literature involved children. Physicians should be made aware of this rare possible outcome to closely monitor their patients and take appropriate measures to prevent such possible adverse effect.

## 1. Introduction

Vincristine (VCR) is widely used in the treatment of acute lymphoblastic leukemia (ALL) [1]. Its antineoplastic effect is attributed to the inhibition of microtubule formation in the miotic spindle causing cell death that may be accompanied by neurological side effects [1, 2]. This neurological toxicity is dependent on both dose and duration of treatment and is characterized by neuropathy, paresthesia, sensory deficits, muscle weakness, and rarely seizures [3]. Vincristine neurotoxic symptoms usually occur within 4 to 10 days of its administration [4] with most symptoms disappearing by about the sixth week after discontinuation of therapy.

Coadministration of azoles (as prophylaxis or treatment of fungal infections) and VCR has been shown to increase VCR neurotoxic effects due to the inhibition of cytochrome P450 (CYP) isoform 3A4, for which VCR is a substrate [1, 5]. Those neurotoxic symptoms usually present as constipation and peripheral neurotoxicity [6, 7]. These symptoms are usually reported after the administration of VCR second dose [1]. Despite the absence of any casual relationship between seizure and coadministration of azoles, few case reports of VCR-induced seizure have been documented after coadministration of fluconazole in an 11-years-old child [8] and coadministration with PSZ in 9- and 4-year-old children [9, 10].

## 2. Case Presentation

This case report was approved by the Medical Research centre of Hamad Medical Corporation, Doha, Qatar. The 19-year-old South west Asian girl, 40 Kg weight and 153 cm tall, was admitted to Al Amal hospital, Doha, Qatar, on June 16, 2010 and was diagnosed with ALL. The induction phase of Berlin Frankfurt Muenster protocol was started on June 23, 2010. The chemotherapy protocol given consisted of (a) Prednisolone (60 mg/m²) 80 mg orally everyday starting from June 23, 2010, (b) VCR (1.5 mg/m²) 2 mg intravenously (i.v.) on days 8, 15, 22, and 29, (c) doxorubicin (30 mg/m²) 40 mg i.v. on days 8, 15, 22, and 29, and (d) L-asparaginase (5000 units/m²) 6500 units i.v. over 60 minutes, days 12, 15, 18, 21, and 24. Intrathecal (IT) Methotrexate 12 mg was administered on days 11 and 18 and IT Hydrocortisone 50 mg on day 18. Posaconazole (PSZ) 200 mg was administered prophylactically three times per day orally starting from July 2, 2010 (day 8).

On July 15, the patient developed septic shock. Next day her hemoglobin dropped to 5.8 g/dL and her stool was found positive for occult blood. As such, the chemotherapy was held and packed RBCs were given.

Five days after the third dose of VCR, July 19 at 10:25 pm, the patient developed her first episode of generalized tonic clonic seizure (GTCS) that lasted for 5 seconds. Then at 10:40 pm of the same day, she developed another episode of GTCS with uprolling of eyes that lasted for 1 minute and aborted by itself. This was followed by postictal loss of consciousness for half an hour. Her blood pressure was 140/96 mmHg, heart rate 100 beats/min, and oxygen saturation of 96% on room air. The patient's laboratory results showed mild hyponatremia, 133 mmol/L; mild hypokalemia, 3.2 mmol/L; phosphorus levels, 0.61 mmol/L; and BUN levels, 5.4 mmol/L. The patient was suspected to have intracranial thrombosis as she was on L-asparaginase. She was transferred to MICU and PSZ was withheld on the same day (July 19).

In MICU, the patient was chemically stable and afebrile, her Blood pressure was 142/77, heart rate was 105 beats/min, oxygen saturation (room air) of 99%, and Glasgow Coma Scale 15/15, conscious, and oriented. The patient had no family history of seizure; her CNS right planter reflex was going upwards otherwise had normal neurological exam. The patient's CT scan was normal with no evidence of intracranial hemorrhage, dermal thrombosis, nor parenchymal lesion.

The patient was put on phenytoin 100 mg i.v. and was admitted back to continue her chemotherapy on July 20. The phenytoin was held and the patient was converted to Levetiracetam oral 500 mg BID until July 23. Then the dose was increased 500 mg am and 1000 mg pm until her discharge. The patient did not have any other episodes of seizure until her discharge on September 26, 2010.

## 3. Discussion

PSZ, a structural analogue of itraconazole, is a new triazole antifungal agent that has been approved for the prophylaxis of invasive Aspergillus and Candida infections in severely immunocompromised patients [11–13]. Those immunocompromised patients include hematopoietic stem cell transplantation patients with graft-versus-host disease or hematologic malignancies patients with prolonged neutropenia from chemotherapy. PSZ has been used for this indication since 2005 in several countries including the USA, Canada, members of the European Union, and Australia [11]. In the USA, PSZ is approved for use in patients ≥13 years old while in Europe it is approved in patients ≥18 years old. PSZ was approved for this indication in Qatar in 2009 and was added in 2010 to formulary of Al-Amal Hospital in Qatar.

VCR, a vinca alkaloid, exhibits peripheral neurotoxicity that involves autonomic nervous system and may be accompanied by syndrome of inappropriate antidiuretic hormone (SIADH) secretion and high blood pressure [9]. Generalized seizure has been reported to result from the hyponatremia associated with SIADH and mostly occurred in patients with seizure disorders [8, 9]. In a study involving 20 pediatric patients, severe CNS toxicity was detected in patients coadministered azoles but not in those administered VCR alone [14].

Up to the authors' knowledge, all of the reported seizures suspected to be caused by the coadministration of PSZ and VCR were in pediatric patients [8–10, 14]. This may be due to the fact that VCR clearance in children is known to be faster than that in adults [15]. As such enzyme inhibitors would cause significantly lower VCR clearance in children with much more aggravated side effects [15]. However, in this study, the authors present the first report of PSZ-VCR drug interaction yielding seizure in a young female adult. Our patient did not have any family history of seizure and her CT scan did not suggest any neurological damage. At the time of seizure, the patient suffered from SIADH symptoms and elevated blood pressure similar to the other cases of VCR toxicity [8–10].

The question that rises is what makes this adult patient more prone to this drug interaction. There are several possible theories which could include lower seizure threshold or altered polymorphic expression of CYP3A5 which can play an important role as a determinant of VCR elimination, systemic exposure, and hence neurotoxicity [16]. However no absolute answer to such question can be provided as this is a retrospective study and has the following limitations. First PSZ and VCR are not therapeutically monitored drugs, as such their plasma or blood concentrations were not readily available. Second is the absence of MRI and EEG data and finally the absence of information on the patient's CYP3A5 allele.

In conclusion, this paper describes a case where a young adult experiences VCR-induced seizure due to PSZ coadministration. This is a novel finding in the sense that all previous case reports pertaining to PSZ-VCR-induced seizure in literature involved children. Physicians should be made aware of this rare possible outcome to closely monitor their patients and take appropriate measures to prevent such possible adverse effect. Thus helping their cancer patients lead better lives. 
